# Optimization of Gamma-Aminobutyric Acid Production by *Lactiplantibacillus plantarum* FRT7 from Chinese Paocai

**DOI:** 10.3390/foods12163034

**Published:** 2023-08-12

**Authors:** Hongying Cai, Xuan Li, Daojie Li, Weiwei Liu, Yunsheng Han, Xin Xu, Peilong Yang, Kun Meng

**Affiliations:** 1 Key Laboratory of Feed Biotechnology of Ministry of Agriculture and Rural Affairs, Institute of Feed Research, Chinese Academy of Agricultural Sciences, Beijing 100081, China; caihongying@caas.cn (H.C.); lixuan01@caas.cn (X.L.); lidaojie0723@163.com (D.L.); liuweiwei@caas.cn (W.L.); hanyunsheng@caas.cn (Y.H.); 82101202006@caas.cn (X.X.); yangpeilong@caas.cn (P.Y.); 2 National Engineering Research Center of Biological Feed, Beijing 100081, China

**Keywords:** *Lactiplantibacillus plantarum* FRT7, GABA synthesis, fermentation parameter optimization, response surface methodology, functional foods

## Abstract

Gamma-aminobutyric acid (GABA) is a widely available non-protein amino acid whose physiological importance goes beyond its role as an inhibitory neurotransmitter in mammals. The GABA synthesis ability of ten strains of *Lactiplantibacillus plantarum* was screened. They produced GABA ranging from 48.19 ± 3.44 to 100.75 ± 1.63 mg/L at 24 h-cultivation. Among them, *Lp. plantarum* FRT7 showed the highest GABA production. Therefore, FRT7 was chosen for GABA yield optimization. A one-factor-at-a-time strategy analysis of the GABA yield of FRT7 was performed, including the culture temperature, incubation time, inoculum volume, initial pH, the initial amount of monosodium glutamate (MSG), and pyridoxal 5’-phosphate (PLP) concentration, based on which the response surface methodology (RSM) was performed. After being cultured in an MRS culture medium supplemented with 3% MSG and 2 mmol/L of PLP at 40 °C with an initial pH of 7.0 for 48 h, the GABA reached a maximum yield of 1158.6 ± 21.22 mg/L. The results showed the experimental value of the GABA yield was in good agreement with the predicted values. Furthermore, the results from the RSM also indicated that the initial MSG addition, PLP concentration, and incubation time were significant variables. These results suggest that *Lp. plantarum* FRT7 has the potential to be a health-beneficial probiotic with commercial capabilities.

## 1. Introduction

Gamma-aminobutyric acid (GABA) is a non-protein amino acid widely found in animals, plants, and microorganisms [1]. GABA is an important inhibitory neurotransmitter in mammals [2]. Intracellular GABA accumulation in some microorganisms can enhance the tolerance to environmental acid stress [3]. The role of GABA in plants includes responding to abiotic and biotic stress factors, maintaining carbon/nitrogen (C/N) balance, and regulating plant development [4]. Furthermore, GABA shows a variety of physiological functions, including enhancing immunity [5], improving sleeplessness and depression [6,7], improving memory functions [8], regulating blood pressure [9], fighting obesity [10], and preventing diabetes [11,12]. Thus, in food and pharmaceuticals, GABA has the potential to be a bioactive ingredient. However, the content of GABA in natural animal and plant-based foods is relatively low [13,14]. Therefore, in recent years, GABA-containing food has attracted a lot of research due to its unique physiological characteristics and wide range of applications.

GABA can be produced by a variety of microorganisms, including yeast [15], fungi [16], and bacteria [17], and their production ability depends on the species and strains [18]. Microbial synthesis of GABA is obtained by the decarboxylation of monosodium glutamate (MSG) under the action of glutamate decarboxylase (GAD: EC 4.1.1.15) [19]. Because its renewable resources, safety, efficiency, and environmental friendliness, and its microbial production method meet the specific needs of the food and pharmaceutical industries, it is considered to be an attractive and promising approach [20]. GABA produced by lactic acid bacteria (LAB) has special physiological activity and commercial potential and can be used as a starter for fermented foods, such as kimchi [21], fermented dairy [22], fermented sausage [23], etc. In recent years, several GABA-producing LAB species have been reported, including *Lp. plantarum* [24], *L. paracasei* [25], *L. brevis* [26], *Streptococcus lactobulus* [27], *L. rhamnosus* [28], etc. Among these, the key species for the production of GABA was found to be *Lp. plantarum* and *L. brevis* [29]. In particular, *Lp. plantarum* has been generally recognized as safe (GRAS), with a qualified presumption of safety (QPS) status [30]. Furthermore, *Lp. plantarum* strains have been used as probiotics due to their immunomodulation properties [31], pathogen suppression [32], modulation of intestinal balance [33], and cholesterol-lowering ability [34]. Therefore, GABA produced by *Lp. plantarum* in appropriate foods can take full advantage of GABA’s health-promoting properties, as well as the *Lp. plantarum* strains themselves.

The ability of LAB to produce GABA widely varies among the strains [35] and is affected significantly by culture conditions, including the substrates, pH, temperature, time, the amount of MSG, etc. [36]. The one-factor-at-a-time (OFAT) strategy is the simplest statistical way to study one key factor, while the interactions among the individual factors are not taken into account, and optimized conditions are often not produced. Response surface methodology (RSM) is a series of mathematical and empirical methods to study the optimal conditions of a multivariable system. The advantage of RSM is that it reduces the number of experiments for evaluating multiple factors and their interactions, thus saving time as well as lowering experimental costs [37]. In this study, ten *Lp. plantarum* strains were screened for GABA-producing ability, and then the strain with the highest production of GABA was chosen to maximize the GABA production by OFAT experiments and the RSM method.

## 2. Materials and Methods

### 2.1. Materials

A de Man, Rogosa, and Sharpe (MRS) agar and MRS broth, used as the selective culture medium for *Lactobacilli*, were purchased from Beijing Solarbio Science & Technology Co., Ltd. (Beijing, China). MSG was purchased from China Huishi Biochemical Reagent Co., Ltd. (Shanghai, China). Anhydrous ethanol was obtained from China National Pharmaceutical Group Corp (Beijing, China). Phthalaldehyde (OPA) was purchased from Shimadzu Analytical China Ltd. (Shanghai, China). Sulfosalicylic acid was bought from Sinopharm Chemical Reagent Co., Ltd. (Shanghai, China). pH analyses were carried out at room temperature using a pH meter (Mettler Toledo^®^- Five Easy, Columbus, OH, USA). GABA production was analyzed using high-performance liquid chromatography (HPLC) (LC-20A, Shimazu, Kyoto, Japan) with a chromatographic column (AJS-02, Shimazu, Kyoto, Japan).

### 2.2. Strains and Microorganism Activation

A total of ten *Lp. plantarum* strains named FRT1 to FRT10 were originally isolated from bean pickles, cabbage pickles, northeast China pickles, yogurt, fermented bean curd soup, fermented fish, Chinese paocai, brewing vinegar, fermented mare’s milk, Poland sourdough, and celery pickles, respectively, identified as *Lp. plantarum* by 16S rRNA sequencing, and preserved in the Key Laboratory of Feed Biotechnology of the Ministry of Agriculture and Rural Affairs. The strains were revived and activated through scribing on the MRS agar plates and then cultured in the MRS liquid culture medium for two generations for each use. The cultivation conditions were as follows: 37 °C, stationary cultivation for 24 h. Afterward, the microbial suspension was prepared with an absorbance value of 1.3 at 600 nm. 

### 2.3. Screening of GABA-Producing LAB

When assessing GABA production, the strains were cultivated in an MRS culture medium (natural pH) containing 3% (*w*/*v*) MSG 30 °C for 24 h in 100 mL flasks containing 30 mL of the medium. The inoculum volume was 3% (*v*/*v*), and then the GABA content in the supernatants was measured after incubation.

### 2.4. Measurement of GABA Content

After 24 h of incubation, the MRS culture medium of *Lp. plantarum* strains from FRT1 to FRT10 was centrifuged at 8000 r/min for 10 min, respectively, and the supernatants were harvested. GABA was purified as follows: we briefly added 1 mL of the supernatant to 1 mL of anhydrous ethanol, oscillated at 200 r/min for 30 min, then centrifuged at 10,000 r/min for 5 min, and then the supernatants were harvested. A total of 2 mL of 5% sulfosalicylic acid was added into 1 mL of the supernatant, ultra-sounded at low temperature for 30 min, and centrifuged at 10,000 r/min for 10 min. The extract was cleaned up by a 0.22-microm filter membrane, and then the GABA concentration was detected by HPLC analysis. OPA was used as the pre-column derivatization reagent. The chromatographic column was the AJS-02 special column for amino acid analysis (C18, 4.6 × 150 mm, 3 μm) (Shimazu, Kyoto, Japan). Disodium phosphate-sodium tetraborate buffer solution (pH 8.2) was the mobile phase A, and methanol–acetonitrile–water (9:9:2) was the mobile phase B. The detection wavelength was 338 nm (first-order amino acid). The column temperature was 50 °C, and the flow rate was 1.6 mL/min. The sample size was 2 μL. The peak area was compared with the corresponding GABA standard to calculate the GABA content. 

GABA in the samples was calculated as X, and its value was expressed as mg/L, calculated according to Formula (1):

X = (Ax × Ci × Vi)/As(1)

X—The content of GABA in the test;
Ax—Peak area of GABA in test solution;
As—Peak area of the GABA standard;
Ci—The concentration of the GABA standard, expressed in (mg/L);
Vi—Dilution ratio of test product solution.

### 2.5. OFAT Strategy for GABA Optimization

*Lp. plantarum* FRT7, with the highest GABA yield among the strains, was selected for the optimization of the culture conditions based on the OFAT approach, which means changing one factor to evaluate the impact of that factor on the GABA yield while the other factors remain unchanged. The optimization parameters included culture temperature (30, 35, 37, 40, and 42 °C), incubation time (12, 24, 36, 48, and 60 h), inoculum volume (0, 1, 2, 3, 4, and 5%), initial pH (4, 4.5, 5, 5.5, 6, 6.5, 7, and 7.5), initial MSG addition (0, 0.5, 1, 1.5, 2, and 3%), and pyridoxal 5’-phosphate concentration (PLP) (0.1, 0.5, 1, 1.5, 2, 4, 6, and 8 mmol/L) on GABA production by *Lp. plantarum* FRT7 under basic fermentation conditions (fermentation time, 24 h, fermentation temperature, 30 °C, 3% of the inoculum volume, 3% of MSG-addition, initial pH 6). The accumulation of GABA in the medium was detected by HPLC. Each experiment was repeated in triplicates.

### 2.6. Experimental Design

Through the analysis and treatment of OFAT experiment results, culture temperature, incubation time, inoculum volume, initial pH, MSG concentration, and PLP concentration were selected for the RSM experiment based on a central composite design (CCD), and the GABA yield was taken as the response values. In this study, threelevels of sixvariables were used to produce a total of 54 combinations (Table 1). Culture temperature (37, 40, 42℃), incubation time (36, 48, 60 h), inoculum volume (3, 4, 5%), initial pH (6.5, 7, 7.5), initial MSG concentration (1, 2, 3%), and PLP concentration (1, 2, 4 mmol/L) were the independent factors for FRT7 to optimize the GABA yield. The 54 treatments were randomized in order to avoid bias, and each experiment was carried out in triplicate. 

### 2.7. Response Surface Methodology (RSM)

The optimal polynomial equation was obtained by fitting the RSM model with the experimental data of the CCD design. Interpreted Design Expert version 8.0.6 trial software (Stat Ease Inc., Minneapolis, MN, USA) was used to perform the data analysis. Analysis of variance (ANOVA), regression analysis, and response surface mapping were used as the main analytical steps to determine the optimal conditions for the production of GABA. Then, the predicted values of the RSM model were compared with actual values to test the model. Finally, the experimental values of the predicted optimal conditions were used as the validating set and compared with the predicted values.

### 2.8. Statistical Analysis

Data were expressed as three repeated means ± standard deviations. One-way ANOVA was used to test the difference between the means, and then Duncan’s multiple-range tests were performed. DesignExpert 8.06 software was used to design the response surface tests, and the results were analyzed. *p* < 0.05 was considered statistically significant. All analyses were carried out using SPSS v.16.0 (SPSS Inc., Chicago, IL, USA).

## 3. Results

### 3.1. Evaluation of GABA-Producing Lp. plantarum

**Figure 1:**
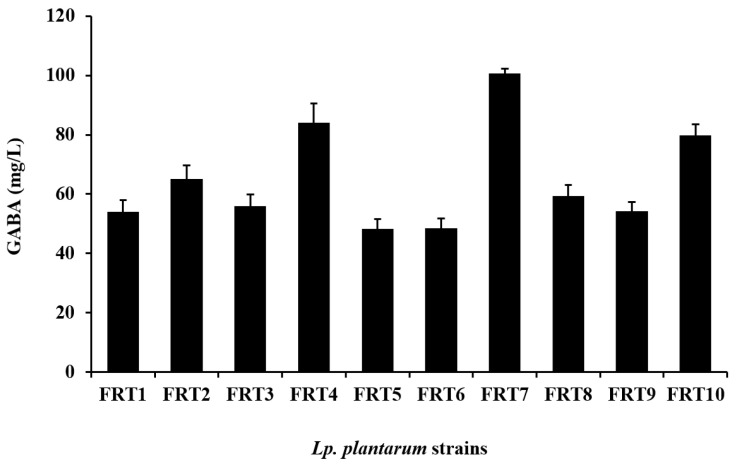
Comparison of GABA production in ten *Lp. plantarum* strains. The strains were cultured in an MRS culture medium containing 3% MSG at 30 °C for 24 h. Data were expressed as the mean ± SD of three replicates.

Ten *Lp. plantarum* strains in this study were evaluated for their GABA-producing ability in an MRS culture medium containing 3% MSG, all of which could produce GABA (Figure 1), and among which *Lp. plantarum* FRT7 from Chinese paocai showed the highest GABA content of 100.75 ± 1.63 mg/L, as measured using HPLC, while the GABA levels of other *Lp. plantarum* strains were lower than 85 mg/L. Therefore, FRT7 was selected for further studies.

### 3.2. Single-Parameter Analysis

#### 3.2.1. Effect of Culture Temperature on the Production of GABA by *Lp. plantarum* FRT7

**Figure 2:**
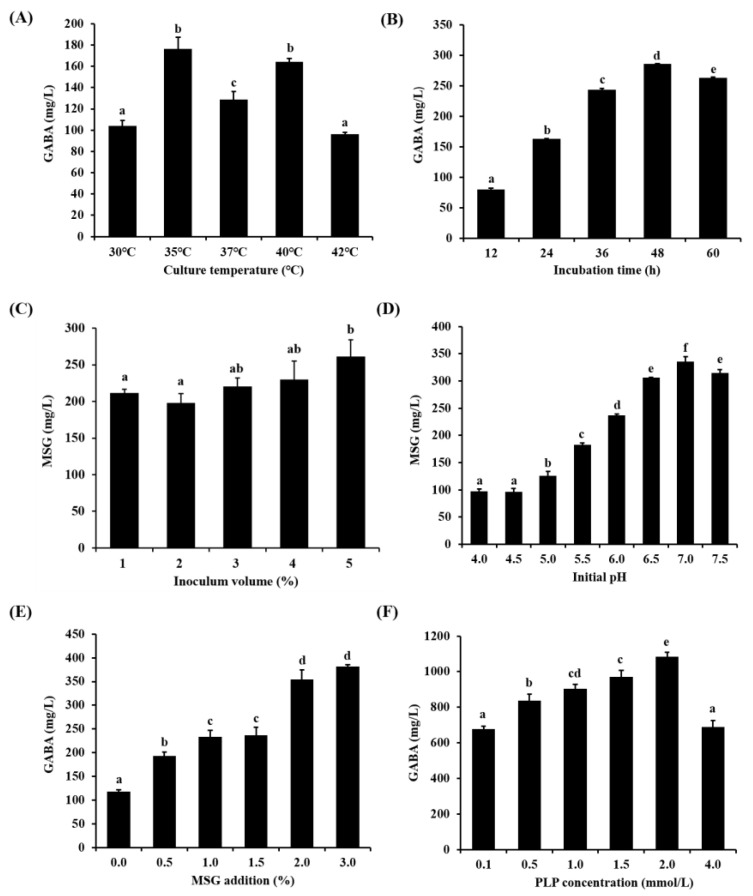
The GABA yield by *Lp. plantarum* FRT7 in the modified MRS culture medium. (**A**) Effect of fermentation temperature on the production of GABA by *Lp. plantarum* FRT7. (**B**) Effects of fermentation time on the production of GABA by *Lp. plantarum* FRT7. (**C**) Effects of inoculum volume on the production of GABA by *Lp. plantarum* FRT7. (**D**) Effects of initial pH on the production of GABA by *Lp. plantarum* FRT7. (**E**) Effect of initial MSG addition on the production of GABA. (**F**) Effect of PLP addition on the production of GABA. The vertical bars represent the SD from 3 replicates. Bars without a common letter had significant differences (*p* < 0.05).

The influence of the culture temperature on the production of GABA was studied in the modified MRS medium under the following conditions (an incubation time of 24 h; an initial pH of 6; an initial MSG addition of 3%; inoculum volume of 3%). As shown in Figure 2A, a high production of GABA was 164.29 ± 3.10 mg/L at 40 °C and 176.51 ± 10.75 mg/L at 35 °C. The yield of the GABA decreased significantly when the temperature increased to 42°. There was no significant difference in the GABA production between 35 °C and 40 °C. A study reported that GAD activity increases with a temperature increase for *L. brevis* 9530 [38]. At the same time, we found that 40 °C had no effect on FRT7 growth. Therefore, 40 °C was selected as the optimal culture temperature for GABA production.

#### 3.2.2. Effect of Incubation Time on the Production of GABA by *Lp. plantarum* FRT7

The influence of the incubation time from 12 to 60 h on the production of GABA in the modified MRS medium was studied under the following conditions (an initial MSG addition of 3%; an inoculum volume of 3%; a culture temperature of 40 °C; an initial pH of 6). From 12 to 48 h, GABA production gradually increased with the increase of the incubation time, among which the maximum GABA yield was 285.30 ± 0.88 mg/L at 48 h, and the GABA production decreased after 48 h (Figure 2B). 

#### 3.2.3. Effect of Inoculum Volume on the Production of GABA by *Lp. plantarum* FRT7

**Figure 3:**
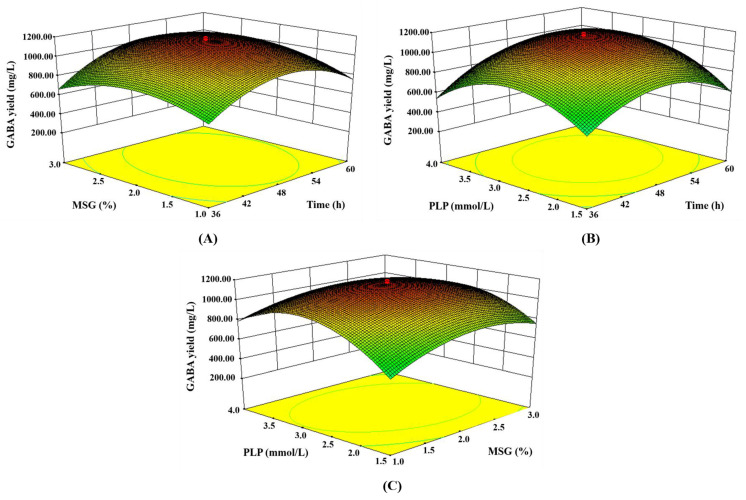
The three-dimensional curved surface diagram shows the influence of different variables on the production of GABA. (**A**) Effects of initial MSG addition and incubation time on GABA yield. (**B**) Effects of PLP concentration and incubation time on GABA yield. (**C**) Effects of PLP concentration and initial MSG addition on GABA yield.

The influence of the inoculum volume from 1 to 5% on the production of GABA in the modified MRS medium was studied under the following conditions (an initial MSG addition of 3%; a culture temperature of 40 °C; an incubation time of 48 h; an initial pH of 6). The inoculum volume was 3%, with approximately 1.8 × 10^10^ CFU. Figure 3C shows the increase in GABA production as the inoculum volume increased from 1 to 5%. The peak GABA yield of 5% of the inoculum volume was 261.48 ± 22.69 mg/L. Considering that there were no significant differences in the inoculum from 3% (220.69 ± 4.09 mg/L) to 5% with the GABA production and saving costs, an inoculum volume of 3% was selected as the optimal condition for the production of GABA in this study. 

#### 3.2.4. Effect of Initial pH on the Production of GABA by *Lp. plantarum* FRT7

The effect of the initial pH from 4 to 7.5 on the production of GABA in the modified MRS medium was studied under the following conditions (an initial MSG addition of 3%; a culture temperature of 40 °C; an incubation time of 48 h; an incubation volume of 3%). As shown in Figure 2D, as the initial pH increased from 4 to 7, GABA production increased and, subsequently, the GABA production decreased. When the initial pH was 7, the GABA concentration reached the maximum value (335.88 ± 9.19 mg/L).

#### 3.2.5. Effect of Initial MSG Addition on the Production of GABA by *Lp. plantarum* FRT7 

The influence of the initial MSG addition from 0 to 3% on the production of GABA in the modified MRS medium was studied under the following conditions (a culture temperature of 40 °C; an incubation time of 48 h; an initial pH of 6; an incubation volume of 3%). As shown in Figure 2E, GABA production was dramatically reduced in the absence of MSG (only 117.92 ± 3.97 mg/L). When the amount of MSG went from 0.5 to 3%, the GABA yield increased in a dose-dependent manner. The maximum GABA production was achieved at 3% with 381.71 ± 4.15 mg/L. 

#### 3.2.6. Effect of PLP Addition Concentration on the Production of GABA by *Lp. plantarum* FRT7

The influence of PLP addition on the production of GABA was assessed with concentrations of 0.1–4 mmol/L in the modified MRS medium under the following conditions (an initial MSG addition of 3%; a culture temperature of 40 °C; an incubation time of 48 h; an initial pH of 6; an incubation volume of 3%). As shown in Figure 2F, the maximum GABA production at the PLP concentration of 2 mmol/L was 1085 ± 24.03 mg/L. However, a further increase in the PLP concentration of more than 2 mmol/L resulted in a decrease in the GABA production from 1085 ± 24.03 to 689.54 ± 37.17 mg/L for *Lp. plantarum* FRT7. The results indicated that PLP addition had a positive impact on GABA production within a certain range. 

### 3.3. Analysis of RSM 

#### 3.3.1. Further Optimization of the Key Factors by RSM

In order to establish the fermentation process model based on single variable optimization, the incubation temperature, incubation time, inoculum volume, initial pH, initial MSG addition, and PLP concentration were selected as valid variables in the response surface design in which the incubation temperature of 40 °C, incubation time of 48 h, inoculum volume of 4%, initial pH of 7, MSG concentration of 2% and PLP concentration of 2 mmol/L were fixed as the central point of the response surface for the response surface analysis, as shown in Table 2.

#### 3.3.2. Response Surface Methodology

Using the experimental data of the CCD’s response surface optimization design, the RSM model was fitted to find the best polynomial equation. Explained Design Expert version 8.0.6 experimental software was employed to analyze the data. Through three main analytical steps, the best model was established to explain the influence of effective factors on the GABA yield.

[GABA] = +1162.04 − 112.13A + 68.88B − 18.88C + 25.39D + 23.86E + 40.71F − 22.83AB − 39.81AC + 6.95AD − 0.14AE + 22.25AF + 4.13BC + 12.07BD + 33.24BE + 48.36BF − 8.91CD − 0.76CE + 14.76CF − 7.69DE + 26.25DF − 55.83EF − 381.95A2 − 255.60B^2^ − 153.91C^2^ − 177.20D^2^ − 161.29E − 283.42F^2^

where A is the culture temperature, B is the incubation time, C is the inoculum volume, D is the initial pH, E is the initial MSG concentration, and G is the PLP concentration.

ANOVA was performed for the GABA production to verify the regression coefficient (Table 3). The “model *p*-value” of the ANOVA was very small (<0.0001), the “lack of fit” was not significant (*p*-value of 0.1097), and it had the appropriate coefficient of determination (R^2^ = 0.98) and adjusted coefficient of determination (R^2^adjusted = 0.96), so the quadratic polynomial model was highly significant and sufficient to represent the actual relationship between the response and the significant variables.

By drawing three-dimensional response surface curves (holding the other variables at the center point), the optimum level of each variable and its interactions with the production of GABA were investigated as a function of two variables. The analysis of variance (Table 2) and three-dimensional plots (Figure 3) show that the incubation time, initial MSG addition amount, and PLP concentration had a significant impact on the production of GABA. Therefore, it was used to develop the model. Figure 3 indicates an effective increase in the production of GABA with the increasing incubation time, initial MSG amount, and PLP concentration up to a certain value and that it then declined after the maximum value.

Figure 3A shows the influence of the initial MSG and incubation time on the production of GABA. The yield of the GABA increased with the increase of the initial MSG addition and incubation time, while the GABA production reached the highest point in the range of 2–2.5% and 48–54 h for the initial MSG addition and incubation time, respectively. In addition, Figure 3A shows that the incubation time’s F value of 37.48 had a significant impact on the production of GABA.

Figure 3B shows the influence of the PLP concentration and incubation time on the production of GABA. The yield of the GABA increased with the increased PLP concentration and incubation time, with the GABA production reaching its highest point when the PLP was added at 2.5–3 mmol/L. In addition, Figure 3B shows that the F value of the PLP was 13.09, which also had an important effect on the GABA yield.

Figure 3C shows the influence of the initial MSG and PLP concentration on the production of the GABA. The yield of the GABA increased with the increase of the initial addition of the MSG and PLP concentration. The initial MSG addition and PLP concentration reached the highest point of GABA production in the range of 2–2.5% and 2.5–3 mmol/L, respectively. This suggests a significant interaction between the initial MSG addition and PLP concentration.

#### 3.3.3. Verification of the Fitted Model and Optimum Point

The actual values of the GABA yield of FRT7 were compared with the predicted values to verify the model. Design Expert software 8.0.6was used to set the best value of the quadratic equation. The experimental conditions fitted to the model were the maximum GABA level predicted by the model (a predicted value of 1179.37 mg/L) at a fermentation temperature of 39.6 °C, a fermentation time of 49.9 h, an inoculum of 3.96%, a pH of 7.0, MSG addition of 2.31%, and PLP concentration of 2.84 mmoL/L. In the actual experiment, the fermentation temperature was adjusted to 40 °C, the fermentation time was 50 h, the inoculum was 4%, the pH was 7.0, the MSG addition was added at 2.3%, the PLP concentration was adjusted to 2.8 mmol/L, and the result showed that the GABA yield of FRT7 was verified to be 1158.6 ± 21.22 mg/L. The predicted value was very close to the actual value of the GABA, and the difference between the two was less than 5%, indicating that the model has high accuracy and reliability.

## 4. Discussion

GABA, as the key metabolite produced by LAB, varied greatly among the strains. The use of LAB as a cell factory for the production of GABA is a fascinating project that opens up broad prospects for the use of GABA and LAB. There is an opportunity to isolate and identify strains that produce GABA for use in functional foods or as probiotics.

In this study, the GABA production ability of ten *Lp. plantarum* strains isolated from Chinese paocai, yogurt, fermented fish, sourdoughs, etc., was investigated. *Lp. plantarum* FRT7, with the highest GABA concentration, was chosen as the most suitable strain for the OFAT optimization of the fermentative parameters to enhance GABA synthesis. LAB’s ability to produce GABA is affected significantly by culture conditions. Temperature has been shown to have a strong effect on GABA production in LAB because both cell growth and GAD activity depend on temperature [18]. *Lp.* plantarum is a mesophilic bacterium with an optimal growth temperature of approximately 37 °C [39]. As shown in Figure 2A, the optimal temperature for the GABA production of *Lp. plantarum* FRT7 was determined at 35 °C. Considering that there were no significant differences between 35 °C and 40 °C and a higher temperature benefits GAD activity, 40 °C was selected as the optimal temperature for further study. Similar results were obtained in previous studies. The production of the highest GABA by *Lp. plantarum* Taj-Apis362 and *Lp. plantarum* Y7 from honeybees and kimchi, respectively, was produced at 37 °C [24,40]. The production of the highest GABA by *Lp. plantarum* DSM19463 was produced between 30 °C and 35 °C [41]. Furthermore, the influence of culture temperature on the production of GABA is mainly due to its effect on GAD activity. Yang et al. reported that when the temperature rose to a certain extent, the GAD activity reached the maximum value, and then the GDA activity would gradually decrease as the temperature further increased [42].

Regarding the effect of incubation time on GABA yield, we observed that the incubation time at 48 h allowed the highest GABA productivity (Figure 2B). However, with a longer incubation time, GABA production decreased; the culture time of the strain was too long, the consumption of the medium was not conducive to the growth of the strain, and the bacteria were in a decline period, which is not conducive to GABA synthesis. Furthermore, the GABA yield decreased in most strains at 72 h compared with 48 h, which may be linked to the degradation by the GABA aminotransferase (GABA-AT, EC 2.6.1.19) [43]. This enzyme degrades GABA into succinic semialdehyde, which is subsequently converted into succinate semialdehyde dehydrogenase (EC 1.2.1.16) for entry into the TCA cycle [44].

Microbial synthesis of GABA is regulated by acidity [29] and is directly related to tolerance to environmental acid stress [19,45]. As shown in Figure 2D, *Lp. plantarum* FRT7 produced a small amount of GABA at an initial of pH 4, and an initial pH of 6.5 to 7 was more helpful to GABA production. Similarly, Thuy et al. [46] reported that the production of GABA reached the maximum level by *Pediococcus pentosaceus* MN12 at a pH of 7. Ko et al. [47] reported that the production of GABA by LAB decreased considerably at an initial pH of 4, with the highest production of GABA at a pH of 6.4. Komatsuzaki et al. [25] reported that an optimal pH value for maintaining LAB GAD activity, and either too high or too low of a pH value, may lead to a partial loss of GAD activity. In microorganisms, GAD is the key enzyme for GABA biosynthesis [48]. GAD has been isolated from several LAB and the biochemical characteristics of some GAD have been characterized [25,49]. The results indicate that although most LAB were active at acidic pH, *Lp. plantarum* FRT7 produced the most GABA in the alkaline pH range, which is beneficial to GAD activity of *Lp. plantarum* FRT7, and this needs further study. 

The role of MSG and PLP in the production of GABA is considered a cofactor of GAD, and GAD converts MSG into GABA [50]. An excessive initial MSG concentration of the fermentation substrate can inhibit cell growth or inhibit the production of GABA, while a low MSG concentration may not meet the needs of the high production of GABA [51]. The effect of MSG concentration on the production of GABA in the range of 0 to 3% was assessed. The optimal concentration of MSG for the production of GABA was 3% (Figure 2E), as the GAD coenzyme, PLP, can influence the production rate of GABA in LAB [19]. As shown in Figure 2F, the GABA contents of *Lp. plantarum* FRT7 increased as the PLP concentration increased to 2 mmol/L and then decreased as the PLP concentration further increased from 2 to 4 mmol/L. These results are consistent with the previous studies that the addition of PLP can improve GAD activity and GABA production [52,53]. The dose–effect relationship between PLP concentration and GABA production needs to be further studied.

The maximum value of GABA production by *Lp. plantarum* FRT7 predicted from the model was 1179.37 mg/L. The experiments were carried out under optimized conditions to verify the predicted results, and the experimental value was 1158.6 ± 21.22 mg/L, which was 115% higher than that in the basal MRS culture medium. Meanwhile, the experimental values of GABA production agreed well with the predicted values. The GABA yield by *Lp. plantarum* Taj-Apis362 and *Lp. plantarum* FBT215 increased from 1.76 to 7.15 mM and 144.02 ± 14.40 to 1812.16 mg/L under optimal culture conditions, respectively [40,54]. These results indicate that *Lp. plantarum* FRT7 has great potential for industry application.

## 5. Conclusions

In this study, ten *Lp. plantarum* strains successfully isolated from Chinese paocai, yogurt, fermented fish, sourdoughs, etc., had GABA production of 48.20 ± 3.44 ~100.74 ± 1.63 mg/L in an MRS culture medium. *Lp. plantarum* FRT7, with the highest GABA yield among the ten *Lp. plantarum* strains, was then selected for the optimization of the culture conditions. The medium composition and culture conditions were optimized by OFAT experiments. The results showed that the optimal conditions for the production of GABA were a culture temperature of 40℃, an incubation time of 48 h, an inoculum volume of 3%, an initial pH of 7, an MSG addition amount of 3%, and a PLP concentration of 2 mmol/L. The results showed that the GABA yield increased significantly from 100.75 ± 1.63 to 1158.6 ± 21.22 mg/L under optimal culture conditions via the OFAT strategy and RSM method. The prediction model is accurate and reliable, which provides guidance for follow-up research. *Lp. plantarum* FRT7 represents a promising strain for the production of GABA in the food industry. However, this still requires the determination of costs and other related factors.

## Figures and Tables

**Table 1:** Factors and levels used in response surface analysis.

Factors	Levels		
	−1	0	1
(A) culture temperature (°C)	37	40	42
(B) incubation time (h)	36	48	60
(C) inoculum volume (%)	3	4	5
(D) initial pH	6.5	7	7.5
(E) MSG concentration (%)	1	2	3
(F) PLP concentration (mmol/L)	1	2	4

**Table 2:** *Lp. plantarum* FRT7 treatment’s incorporation and responses in the CCD with actual and predicted values of the production of GABA.

Run	Culture Temperature (℃)	Incubation Time (h)	Inoculum Volume (%)	pH	MSG Concentration (%)	PLP Concentration (mmol/L)	Actual GABA (mg/L)	Predicted GABA (mg/L)
1	0	−1	−1	0	−1	0	495.79 ± 14.03	553.98
2	0	0	1	−1	0	1	604.77 ± 35.52	541.36
3	0	0	−1	−1	0	−1	551.66 ± 24.03	532.39
4	−1	1	0	1	0	0	555.92 ± 37.05	581.63
5	0	0	0	0	0	0	1179.39 ± 24.98	1162.04
6	0	0	0	0	0	0	1190.21 ± 39.01	1162.04
7	0	1	0	0	−1	−1	274.63 ± 17.05	328.61
8	1	−1	0	1	0	0	193.7 ± 25.15	209.39
9	−1	0	0	1	−1	0	586.33 ± 41.55	555.86
10	−1	0	1	0	0	−1	406.06 ± 16.24	442.59
11	1	0	0	−1	−1	0	263.19 ± 16.35	265.71
12	0	0	0	0	0	0	1148.86 ± 32.07	1162.04
13	0	−1	−1	0	1	0	540.41 ± 45.10	536.74
14	0	−1	0	0	−1	1	455.2 ± 15.09	450.41
15	1	1	0	−1	0	0	200.08 ± 19.79	236.79
16	−1	−1	0	1	0	0	390.07 ± 13.20	374.07
17	−1	0	0	1	1	0	611.71 ± 41.66	588.47
18	−1	0	0	−1	1	0	559.62 ± 27.21	566.96
19	0	0	0	0	0	0	1186.71 ± 32.45	1162.04
20	−1	0	0	−1	−1	0	509.63 ± 40.5	503.59
21	1	0	0	1	−1	0	332.41 ± 9.15	345.78
22	0	1	0	0	−1	1	568.81 ± 26.12	618.39
23	0	1	0	0	1	−1	619.11 ± 25.88	554.47
24	0	0	1	−1	0	−1	412.36 ± 20.89	482.92
25	0	−1	0	0	1	−1	427.09 ± 16.12	446.94
26	−1	0	−1	0	0	1	412.6 ± 19.98	437.65
27	1	−1	0	−1	0	0	173.84 ± 3.96	168.84
28	0	0	−1	1	0	−1	545.36 ± 27.21	548.49
29	1	0	0	−1	1	0	318.78 ± 25.07	328.54
30	−1	0	1	0	0	1	472.55 ± 7.89	509.02
31	0	−1	0	0	−1	−1	430.75 ± 11.20	354.04
32	0	1	1	0	1	0	690.45 ± 45.23	701.70
33	1	1	0	1	0	0	313.76 ± 21.00	325.62
34	1	0	1	0	0	1	202.99 ± 15.51	249.66
35	0	1	−1	0	1	0	715.84 ± 30.22	732.72
36	0	−1	0	0	1	1	304.53 ± 29.28	319.99
37	1	0	1	0	0	−1	179.55 ± 3.35	94.22
38	0	−1	1	0	−1	0	456.94 ± 12.47	509.49
39	1	0	−1	0	0	1	434.32 ± 20.58	337.51
40	1	0	−1	0	0	−1	217.31 ± 15.19	241.11
41	0	0	0	0	0	0	1100.2 ± 31.24	1162.04
42	1	0	0	1	1	0	351.1 ± 5.89	377.85
43	−1	−1	0	−1	0	0	393.89 ± 21.30	361.32
44	0	0	0	0	0	0	1166.85 ± 25.60	1162.04
45	0	0	1	1	0	1	667.84 ± 15.09	626.83
46	0	1	1	0	−1	0	654.79 ± 21.34	589.02
47	0	1	0	0	1	1	613.66 ± 35.04	620.93
48	0	0	1	1	0	−1	438.92 ± 27.39	463.40
49	0	−1	1	0	1	0	512.22 ± 35.71	489.21
50	−1	1	0	−1	0	0	557.01 ± 20.72	520.61
51	0	0	−1	1	0	1	663.17 ± 35.81	652.88
52	0	0	−1	−1	0	1	495.99 ± 29.11	531.79
53	−1	0	−1	0	0	−1	416.65 ± 13.45	430.26
54	0	1	−1	0	−1	0	663.43 ± 27.82	617.00

Notes: Values are means of three replicates ± SDs. The Box–Behnken design was used in the experiment, and the predicted value was obtained by regression equation analysis.

**Table 3:** Analysis of variance for GABA production by *Lp. plantarum* FRT7.

Source	SS	DF	MS	F Value	Prob > F	
Model	3.66 × 10^6^	27	1.36 × 10^5^	44.66	<0.0001	significant
A-A	3.02 × 10^5^	1	3.02 × 10^5^	99.31	<0.0001	
B-B	1.14 × 10^5^	1	1.14 × 10^5^	37.48	<0.0001	
C-C	8553.77	1	8553.77	2.82	0.1053	
D-D	15,477.24	1	15,477.24	5.09	0.0326	
E-E	13,662.24	1	13,662.24	4.5	0.0437	
F-F	39,770.41	1	39,770.41	13.09	0.0013	
AB	4171.04	1	4171.04	1.37	0.2519	
AC	12,676.3	1	12,676.3	4.17	0.0513	
AD	772.84	1	772.84	0.25	0.6183	
AE	0.15	1	0.15	4.89 × 10^-5^	0.9945	
AF	3960.95	1	3960.95	1.3	0.2639	
BC	136.21	1	136.21	0.045	0.834	
BD	1165.24	1	1165.24	0.38	0.5411	
BE	17,677.7	1	17,677.7	5.82	0.0232	
BF	18,705.65	1	18,705.65	6.16	0.0199	
CD	634.57	1	634.57	0.21	0.6515	
CE	4.64	1	4.64	1.53 × 10^-3^	0.9691	
CF	3485.72	1	3485.72	1.15	0.294	
DE	472.94	1	472.94	0.16	0.6964	
DF	5511.98	1	5511.98	1.81	0.1896	
EF	24,935.91	1	24,935.91	8.21	0.0081	
A^2	1.50 × 10^6^	1	1.50 × 10^6^	493.91	<0.0001	
B^2	6.72 × 10^5^	1	6.72 × 10^5^	221.19	<0.0001	
C^2	2.44 × 10^5^	1	2.44 × 10^5^	80.2	<0.0001	
D^2	3.23 × 10^5^	1	3.23 × 10^5^	106.3	<0.0001	
E^2	2.68 × 10^5^	1	2.68 × 10^5^	88.07	<0.0001	
F^2	8.26 × 10^5^	1	8.26 × 10^5^	271.95	<0.0001	
Residual	78,991.76	26	3038.14			
Lack of Fit	73,267.54	21	3488.93	3.05	0.1097	not significant
Pure Error	5724.21	5	1144.84			
Cor Total	3.74 × 10^6^	53				

Notes: SS, sum of squares; MS, mean squares; A, culture temperature; B, incubation time; C, inoculum volume; D, pH; E, initial MSG addition; G, PLP concentration.

## Data Availability

The datasets generated for this study are available on request to the corresponding author.
